# P-11. Evolution of Gram-Negative Rod Bacteremia Management in Hematology and Oncology Patients

**DOI:** 10.1093/ofid/ofaf695.242

**Published:** 2026-01-11

**Authors:** Cameron Rehmani, David R Ha, William Alegria, Alex Zimmet, Sa Shen, Ariadna Garcia, Niaz Banaei, Marisa Holubar

**Affiliations:** Stanford Health Care, Palo Alto, CA; Stanford Health Care, Palo Alto, CA; Stanford Health Care, Stanford University School of Medicine, Stanford, California; Stanford Healthcare, Stanford University School of Medicine, Palo Alto, California; Quantitative Sciences Unit, Stanford, California; Stanford Health Care, Palo Alto, CA; Stanford University School of Medicine, Palo Alto, CA; Stanford University School of Medicine, Palo Alto, CA

## Abstract

**Background:**

Managing uncomplicated Gram-negative rod bacteremia (uGNB) with shorter durations of therapy and oral antibiotic transition has been well-established by randomized trials and large retrospective studies. However, immunocompromised patients, like hematology and oncology patients, are often underrepresented in such trials. Our goal was to describe uGNB management over a contemporary 5-year period in this population.

**Figure:**
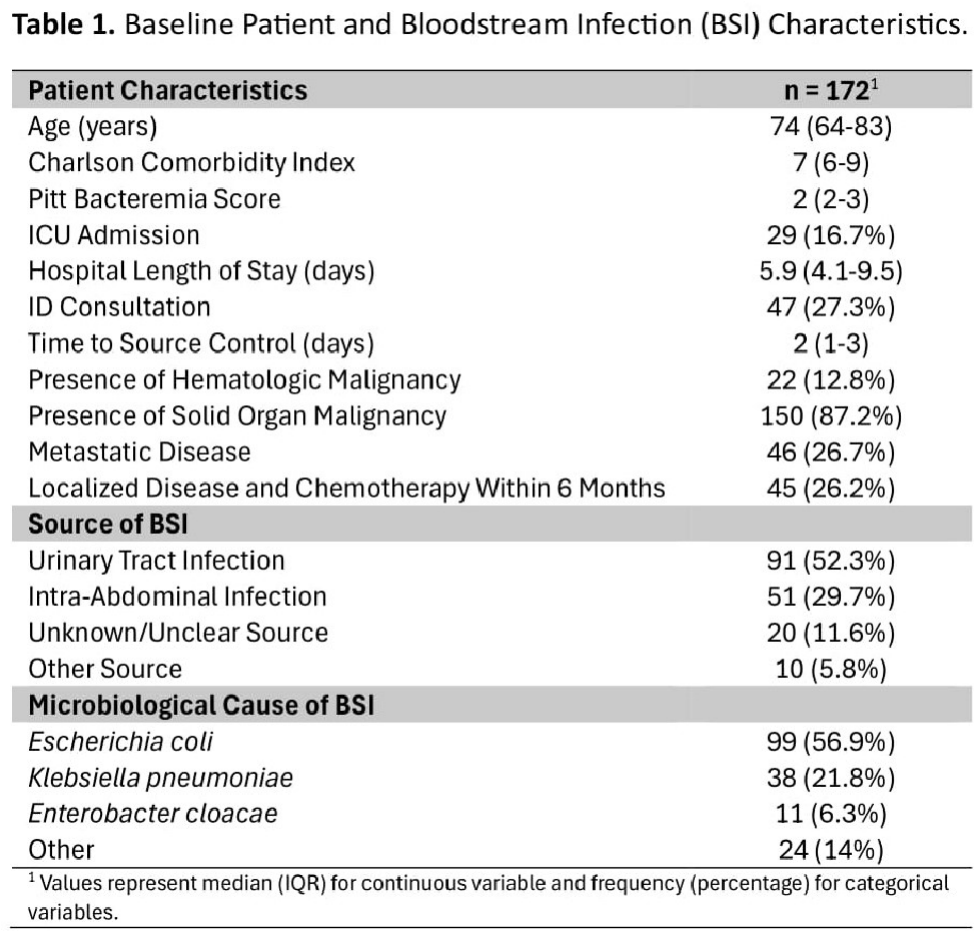

**Figure:**
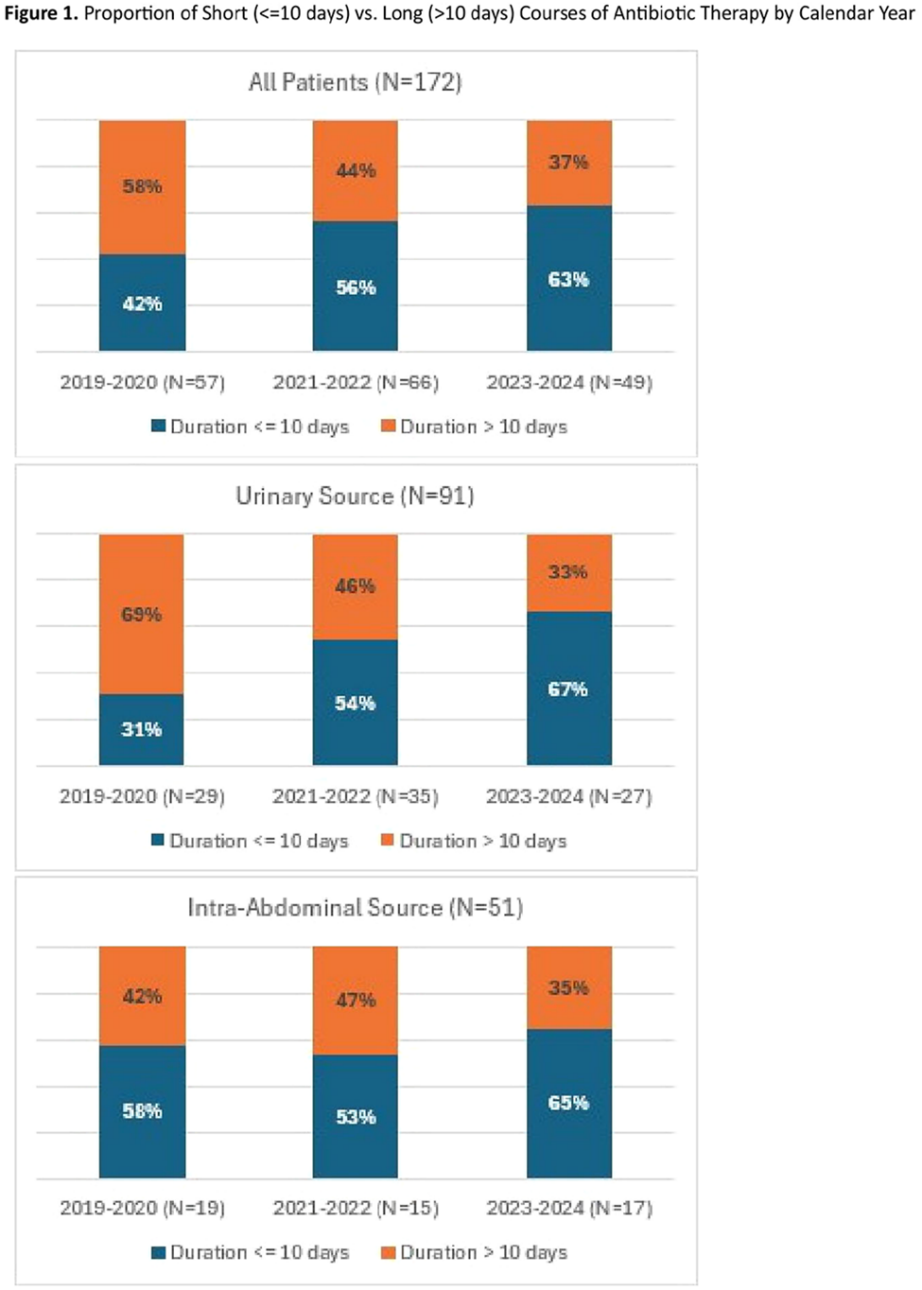

**Methods:**

This single-center, retrospective, study included adults with an active hematologic or solid malignancies and monomicrobial Enterobacterales bloodstream infection managed at Stanford Hospital between 2019 and 2024. Duration was categorized as short (≤10 days) or long ( >10 days). Exclusion criteria included bacteremia secondary to endocarditis, meningitis, or osteomyelitis, uncontrolled source of infection, failure to receive at least one active antibiotic, resistance to carbapenems, allogenic bone marrow transplant, and transition to hospice care or death within 14 days of culture.

**Figure:**
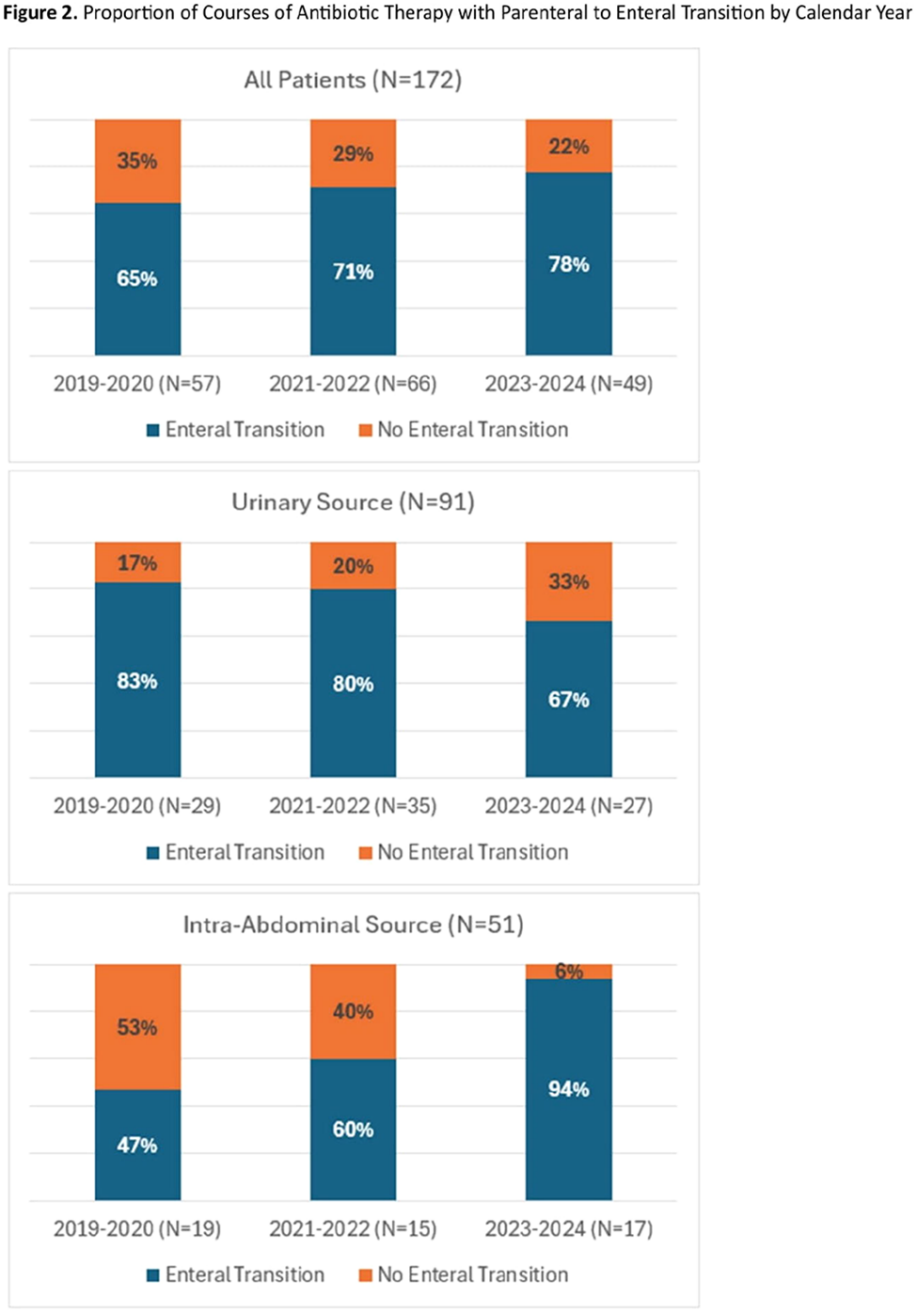

**Results:**

172 patients were included. Primary sources of infection included urinary (52.3%) and intra-abdominal (26.7%) with the most common organism being Escherichia coli (56.9%). Most had a solid organ malignancy (87.2%); 26.7% of these had metastatic disease and 26.2% had localized disease with chemotherapy within 6 months. The prevalence of short antibiotic duration increased from 42% to 63% (Figure 1) and oral antibiotic transition increased from 65% to 78% (Figure 2). When classified by the most common indications, increase in short duration was mainly driven by urinary tract infections and oral antibiotic transition was mainly driven by intra-abdominal infections.

**Conclusion:**

Despite major comorbidity, contemporary uGNB antibiotic treatment paradigms, namely, shorter duration and oral transition, were increasingly employed in hematology oncology patients.

**Disclosures:**

All Authors: No reported disclosures

